# Common polymorphisms of the *hOGG1, APE1* and *XRCC1* genes correlate with the susceptibility and clinicopathological features of primary angle-closure glaucoma

**DOI:** 10.1042/BSR20160644

**Published:** 2017-05-17

**Authors:** Kun Zeng, Bo Zhong, Min Fang, Xiao-Li Shen, Li-Na Huang

**Affiliations:** 1 Shenzhen Key Laboratory of Ophthalmology, Shenzhen Eye Hospital, Shenzhen 518000, P.R. China; 2 Department of Stomatology, Shenzhen Second People’s Hospital, Shenzhen 518035, P.R. China

**Keywords:** APE1, Clinicopathological features, hOGG1, Primary angle-closure glaucoma, Polymorphism, Susceptibility, XRCC1

## Abstract

The present case study aims to elucidate the correlation between the human 8-hydroxyguanineglycosylase (*hOGG1*), *APE1* and X-ray repair cross-complementing gene 1 (*XRCC1*) gene polymorphisms to the susceptibility and clinicopathological features of primary angle closure glaucoma (PACG) in a Chinese Han population. Blood samples were obtained from 258 PACG patients (case group) and 272 healthy volunteers (control group). PCR with sequence-specific primer (PCR-SSP) was used to determine the allele frequencies and genotype distributions of the *hOGG1, APE1* and *XRCC1* genes. The risk factors of PACG were determined using logistic regression analysis. The results indicated that *hOGG1* Ser326Cys, *APE1* Asp148Glu and *XRCC1* Arg399Gln polymorphisms were correlated with the risk of PACG. Furthermore, there were thicker corneas, higher intraocular pressure (IOP) and a shorter axial length in patients carrying the mutant genotypes of *hOGG1* Ser326Cys (Ser/Cys + Cys/Cys), *APE1* Asp148Glu (Asp/Glu + Glu/Glu) and XRCC*1* Arg399Gln (Arg/Gln + Glu/Glu) than those carrying the corresponding wild-type genotypes. According to the logistic regression analysis, Asp148Glu and Arg399Gln polymorphisms, a short axial length and high IOP are major risk factors for PACG. These findings reveal that *hOGG1* Ser326Cys, *APE1* Asp148Glu and *XRCC1* Arg399Gln polymorphisms are correlated with the risk and clinicopathological features of PACG in a Chinese Han population.

## Introduction

Glaucoma is characterized by a progressive degeneration of retinal ganglion cells (RGCs) and optic nerve axons. It also causes damage to the visual field and has been listed as the second highest cause of blindness worldwide [1]. Globally, it is estimated that 60 million people suffer from glaucomatous optic neuropathy and glaucoma is the cause of blindness in 8.4 million people [2]. Nowadays, ethnicity, gender and age are identified as risk factors for primary angle closure glaucoma (PACG) [3]. Although PACG is a leading cause of irreversible blindness, visual ability can be maintained if early and proper treatment is adopted [4]. According to recent reports, gene polymorphism is an important factor in determining an individual’s disease susceptibility, phenotype and treatment response. Furthermore, gene polymorphism is reported to be strongly correlated with glaucoma susceptibility [5,6].

Human 8-hydroxyguanineglycosylase (*hOGG1*) is a DNA-repair enzyme which can target and remove 8-dihydro-8-oxoguanine (8-OH-G) to repair damaged DNA [7]. The *APE1* gene is located on chromosome 14q11.2-q12 and the amino acid alterations at codon 148 (Asp/Glu) in exon 5 is a common research topic. This polymorphism may be related to ionizing radiations hypersensitivity [8]. *APE1* is capable of hydrolysing 3′-blocking fragments from oxidized DNA and is involved in the creation of 3′-hydroxyl nucleotide termini, is a crucial factor of ligation at single- or double-strand breaks and DNA repair synthesis [8]. X-ray repair cross-complementing gene 1 (XRCC1) has been shown to contribute to the repair of damaged DNA [9]. At present, multiple genes and genetic loci that lead to glaucoma have been found, most of which are related to primary open angle glaucoma (POAG) [10]. There are reports that suggest an association among the *hOGG1, APE1* and *XRCC1* genes and a susceptibility to oesophageal, breast and bladder cancer [11–13]. hOGG1, APE1 and XRCC1 initiates base excision repair (BER) [14–16] and it plays a role in the development of POAG [17]. The present study aims to explore the potential association of *hOGG1, XRCC1* and *APE1* gene polymorphisms with the susceptibility and clinicopathological features of PACG in a Chinese Han population. We hope to provide a theoretical foundation for the early diagnosis of PACG.

## Materials and methods

### Study subjects

Han PACG patients (*n*=258) receiving treatment from February 2008 to October 2014 in the Department of Ophthalmology at Shenzhen Eye Hospital were selected as the case group (141 males and 117 females aged between 37 and 83 years old with an average age of 59.3 ± 6.7 years). Among them, there were 151 acute angle-closure glaucoma (AACG) patients and 107 chronic angle closure glaucoma (CACG) patients. Meanwhile, 272 healthy volunteers were recruited as the control group. There was no significant difference in age, gender or ethnicity between the case and control groups. The inclusion criteria are based on the diagnostic criteria for PACG issued by the International Society of Geographical and Epidemiological Ophthalmology (ISGEO) [18]: (i) primary angle closure suspect (PACS): an eye in which appositional contact between the peripheral iris and posterior trabecular meshwork is considered possible, (ii) primary angle closure (PAC): an eye with an occludable angle and features indicating that trabecular obstruction by the peripheral iris has occurred. The optic disc does not have glaucomatous damage, (iii) PACG: PAC together with evidence of glaucomatous optic neuropathy. The exclusion criteria were: (i) patients with other eye diseases that may lead to a damaged optic nerve or retina, (ii) patients with a family history of genetic disease other than PACG, (iii) patients with secondary glaucoma or open-angle glaucoma, (iv) patients with various chronic diseases, tumours or have a poor liver and kidney functioning. This research was approved by ethics committee of Shenzhen Eye Hospital and informed consent was signed by all the participants.

### Single nucleotide polymorphism screening

The single nucleotide polymorphism (SNPs) of *hOGG1, APE1* and *XRCC1* genes in a Chinese Han population were obtained from the HapMap database. The data were imported into the Haploview Software (version: 4.2) to select tag SNPs based on the following criteria: *r*^2^>0.8 and minor allele frequency (MAF) >0.05. The confidence interval method of linkage disequilibrium value (D’ value), the adjacent SNP of D′ value 95% confidence interval (CI) between 0.70 and 0.98 was classified into the same haplotype block. The tag SNP Ser326Cys was selected from the *hOGG1* gene, Asp148Glu from the *APE1* gene and Arg399Gln from the *XRCC1* gene. The SNPs site variation information is shown in Table 1.

**Table 1:** Variation of *hOGG1, APE1* and *XRCC1* SNPs

Gene	dbSNP	Function	Alleles	Allele frequency (CHB)
*hOGG1*	Ser326Cys	Missense	Ser/Cys	A: 0.7050, B: 0.2950
*APE1*	Asp148Glu	Missense	Asp/Glu	A: 0.5665, B: 0.4335
*XRCC1*	Arg399Gln	Missense	Arg/Gln	A: 0.2317, B: 0.7683

CHB, HapMap database for Han Chinese in Beijing.

### SNP sequencing

Five millilitres of elbow vein blood was drawn from all the fasting subjects, anticoagulated with EDTA and preserved in a refrigerator at –70°C. Genomic DNA from the peripheral venous blood was extracted using the phenol–chloroform extraction method. SNP sequencing was performed using the TaqMan probe method. Multiple PCR with the sequence-specific primer (PCR-SSP) method was used to amplify *hOGG1, APE1* and *XRCC1* genotyping. PCR primers were designed using the Primer Premier Software (version: 5.0) and synthesized at the Beijing Institute of Genomics (Beijing, China). The sequence of each primer is shown in Table 2. The PCR reaction system was 25 μl in total, containing 2 μl of DNA template and 0.2 μl of Taq DNA polymerase (Promega Corp., Madison, WI, U.S.A.). The PCR was conducted using a PCR instrument (S1000, Bio–Rad, U.S.A.) and the reaction conditions were as follows: 30 cycles of predenaturing at 95°C for 10 min, denaturing at 95°C for 1 min, annealing at 64°C for 1 min, extension at 72°C for 1 min followed by a final extension at 72°C for 5 min. After PCR, the PCR product was added into wells with 2% agarose gel. The PCR products underwent electrophoresis at a voltage of 250V for 20 min and the gel imaging system was used to detect and photograph the products.

**Table 2:** Primer sequences of *hOGG1, APE1* and *XRCC1* gene polymorphisms

Gene	Primer sequence	Product length
*hOGG1*	Forward: 5′-TTGATGGGTCACAGAAGGG-3′	552 bp
	Reverse: 5′-TGAGGTAGTCACAGGGAGGC-3′	
*APE1*	Forward: 5′-GAGGAATTGG AGCGTTAACTGT-3′	168 bp
	Reverse: 5′-GCTTATTCACCACGAAIAGCC-3′	
*XRCC1*	Forward: 5′-TCCCTGCGCCGCTGCAGTTTCT-3′	447 bp
	Reverse: 5′-TGGCGTGTGAGGCCTTACCTCC-3′	

### Statistical analysis

Statistical software (version: SPSS19.0) was used for all the data analysis. Measurement data are expressed as mean ± S.D. ($\bar{x}$ ± s) and was examined by the *t* test. Count data are expressed as a percentage or ratio and was tested with the χ^2^ or Fisher’s exact tests. The χ^2^ test was used to analyse whether the genotype distributions of *hOGG1, APE1, XRCC1* and the control group were in accordance with the Hardy–Weinberg equilibrium. Logistic regression analysis was applied to analyse the influence factors of PACG. The *P*-value was two-sided and a *P*<0.05 indicated statistical significance.

## Results

### Genotyping of *hOGG1, APE1* and *XRCC1* polymorphisms

**Figure 1:**
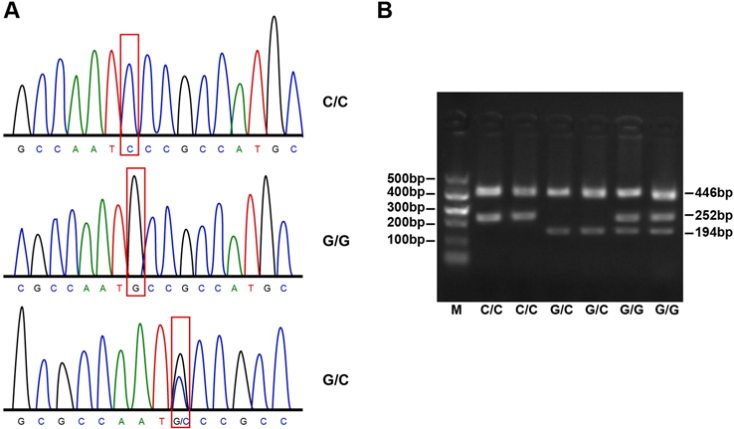
Agarose gel electrophoresis and PCR products of hOGG1 Ser326Cys (Ser/Cys).SNP Ser326 of *hOGG1* gene exhibited fragment 446 bp after amplification, which caused three different fragments (194, 252 and 446 bp). The homozygous wild-type (Ser/Ser) was 252 and 446 bp, the homozygous mutation (Cys/Cys) was 194 and 446 bp and heterozygote (Ser/Cys) was 194, 252 and 446 bp.

**Figure 2:**
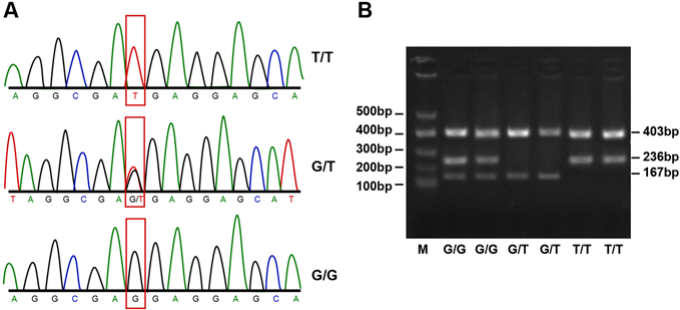
Agarose gel electrophoresis and PCR products of APE1 Asp148Glu (Asp/Glu). SNP 148 of *APE1* gene exhibited fragment 403 bp after amplification, which caused three different fragments (167, 236 and 403 bp). The homozygous wild-type (Asp/Asp) was 236 and 403 bp, the homozygous mutation (Glu/Glu) was 194 and 446 bp and heterozygote (Asp/Glu) was 167, 236 and 403 bp.

**Figure 3:**
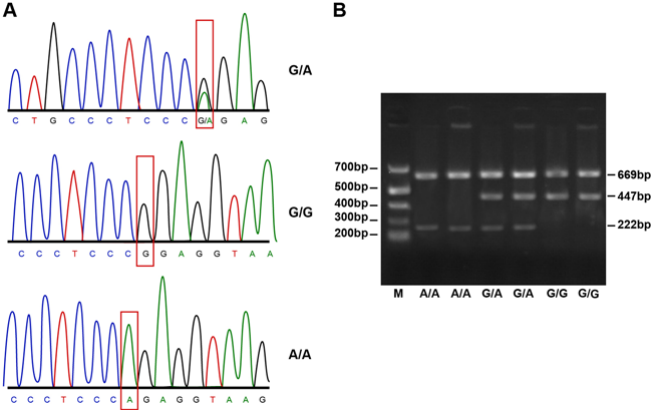
Agarose gel electrophoresis and PCR products of XRCC1 Arg399Gln (Arg/Gln). SNP 399 of *XRCC1* gene exhibited fragment 447 bp after amplification, which caused three different fragments (447, 222 and 669 bp). The homozygous wild-type (Arg/Arg) was 447 and 669 bp, the homozygous mutation (Gln/Gln) was 463 and 669 bp and heterozygote (Arg/Gln) was 222, 447 and 669 bp.

SNPs of *hOGG1, APE1* and *XRCC1* were analysed using multiple PCR. Identifying the specific alleles on each primer allowed for PCR amplified fragments (which were digested by enzymes of the four polymorphic sites) to be obtained. The genotypes gained by DNA sequencing were the same as those gained through the PCR-SSP method (Figures 1–3).

### Hardy–Weinberg equilibrium testing of the genotype distributions of hOGG1, APE1 and XRCC1 gene polymorphisms in the control group

The genotype frequency of the control group was in accordance with the Hardy–Weinberg equilibrium. After the Hardy–Weinberg equilibrium testing, the genotype frequencies of the *hOGG1, APE1* and *XRCC1* genes in the control group showed no significant difference from each other (all *P*>0.05). This indicates that the sample was a good representation of the population.

### Comparison of clinicopathological characteristics between the case and control groups

As shown in Table 3, there was no significant difference in gender, age and diastolic pressure between the case and control groups (all *P*>0.05). However, patients in the case group exhibited a remarkably lower eyesight ability, shorter axial length, higher systolic pressure and intraocular pressure (IOP) and thicker cornea than the control group (all *P*<0.05).

**Table 3:** Comparison of clinicopathological features between the case group and the control group

Clinicopathological features	Case group	Control group	χ^2^/t	*P*
Gender (male/female)	141/117	158/114	0.636	0.425
Age (years)	59.3 ± 6.7	56.7 ± 7.1	1.832	0.068
Diseased eye (both eyes/one eye)	116/142	-		
Eyesight	0.5 ± 0.1	0.6 ± 0.1	11.51	<0.001
Blood pressure				
Systolic pressure (mmHg)	140.6 ± 8.5	131.4 ± 7.6	13.15	<0.001
Diastolic pressure (mmHg)	86.1 ± 6.5	85.7 ± 6.4	0.714	0.476
Eye condition				
Axial length (mm)	22.5 ± 1.3	24.7 ± 1.5	13.11	<0.001
Cornea thickness (μm)	544.3 ± 30.5	540.1 ± 30.2	2.127	0.034
IOP (mmHg)	24.3 ± 7.0	19.8 ± 5.3	7.393	<0.001

### Allele frequencies and genotype distributions of *hOGG1* Ser326Cys, *APE1* Asp148Glu and *XRCC1* Arg399Gln in the case and control groups

**Figure 4:**
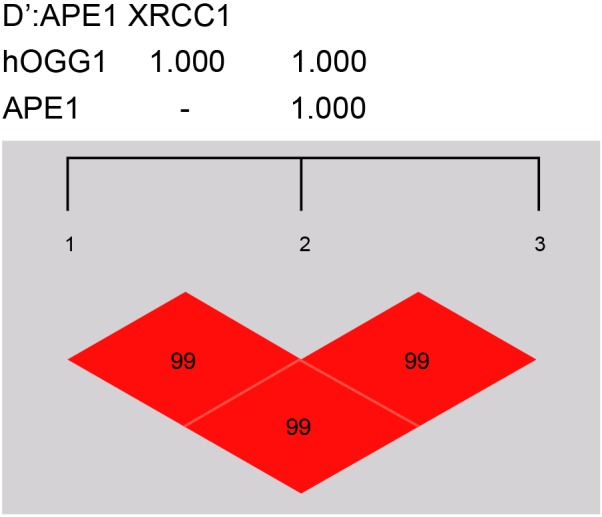
Linkage analyses of the polymorphic loci of the case group and the control group.

Allele and genotype frequency distributions of *hOGG1* Ser326Cys, *APE1* Asp148Glu and *XRCC1* Arg399Gln in the case and control groups are shown in Table 4. The genotype distributions of the case and control groups were tested through linkage disequilibrium. The results show that *hOGG1* Ser326Cys and *APE1* Asp148Glu had D’ and *r*^2^ values of 0.991 and 0.824 respectively; *hOGG1* Ser326Cys and *XRCC1* Arg399Gln had D’ and *r*^2^ values of 0.993 and 0.871 respectively; *APE1* Asp148Glu and *XRCC1* Arg399Gln had D’ and *r*^2^ values of 0.995 and 0.875 respectively (Figure 4). The risk of PACG is associated with *hOGG1* Ser326Cys (Ser/Ser compared with Cys/Cys: odds ratio (OR) =1.788, *P*=0.018; Ser/Ser compared with (Ser/Cys + Cys/Cys): OR =1.821, *P*=0.002; Serine compared with Cysteine: OR =1.367, *P*=0.011). *APE1* Asp148Glu is associated with PACG risk (Asp/Asp compared with Glu/Glu: OR =1.833, *P*=0.021; Asparagine compared with Glutamic acid: OR =1.323, *P*=0.023). *XRCC1* Arg399Gln is also associated with PACG risk (Arg/Arg compared with Glu/Glu; OR =2.491, *P*=0.008; Arg/Arg compared with (Arg/Gln + Glu/Glu): OR =1.796, *P*=0.001; Arginine compared with Glutamic acid: OR =1.574, *P*=0.001).

**Table 4:** Allele frequencies and genotype distributions of *hOGG1* Ser326Cys, *APE*1 Asp148Glu and *XRCC1* Arg399Gln between the case and control groups

SNPs	Genotype	Case group (*n*=258)	Control group (*n*=272)	χ^2^	OR (95% CI)	*P*-value
*hOGG1* Ser326Cys	Ser/Ser	58 (22.5%)	94 (34.6%)	Ref.		
	Ser/Cys	136 (52.7%)	120 (44.1%)	8.567	1.837 (1.220–2.765)	0.003
	Cys/Cys	64 (24.8%)	58 (21.3%)	5.604	1.788 (1.103–2.899)	0.018
	Ser/Cys + Cys/Cys	200 (77.5%)	178 (65.4%)	9.444	1.821 (1.240–2.675)	0.002
	Serine	252 (48.8%)	308 (56.6%)	Ref.		
	Cysteine	264 (51.2%)	236 (43.4%)	6.433	1.367 (1.073–1.742)	0.011
*APE1* Asp148Glu	Asp/Asp	44 (17.1%)	61 (22.4%)	Ref.		
	Asp/Glu	136 (52.7%)	152 (55.9%)	0.876	1.240 (0.790–1.948)	0.349
	Glu/Glu	78 (30.2%)	59 (21.7%)	5.371	1.833 (1.096–3.066)	0.021
	Asp/Glu + Glu/Glu	214 (82.9%)	211 (77.6%)	2.405	1.406 (0.913–2.166)	0.121
	Asparagine	224 (43.4%)	274 (50.4%)	Ref.		
	Glutamic acid	292 (56.6%)	270 (49.6%)	5.145	1.323 (1.039–1.685)	0.023
*XRCC1* Arg399Gln	Arg/Arg	103 (39.9%)	148 (54.4%)	Ref.		
	Arg/Gln	129 (50.0%)	109 (40.1%)	8.492	1.701 (1.189–2.433)	0.004
	Glu/Glu	26 (10.1%)	15 (5.5%)	7.157	2.491 (1.257–4.934)	0.008
	Arg/Gln + Glu/Glu	155 (60.1%)	124 (45.6%)	11.150	1.796 (1.272–2.536)	0.001
	Arginine	335 (64.9%)	405 (74.4%)	Ref.		
	Glutamic acid	181 (35.1%)	139 (25.6%)	11.400	1.574 (1.209–2.050)	0.001

Ref, control.

### Correlation of *hOGG1* Ser326Cys, *APE1* Asp148Glu and *XRCC1* Arg399Gln polymorphisms with the clinicopathological features of PACG patients

There was no difference in gender, age, diseased eye, eyesight and blood pressure among the different polymorphisms of *hOGG1, APE1* and *XRCC1* (all *P*>0.05). However, patients carrying the mutation genotype of *hOGG1* Ser326Cys (Ser/Cys + Cys/Cys) had thicker corneas, higher IOP and shorter axial lengths than those with the Ser/Ser wild-type genotype of *hOGG1* Ser326Cys. Patients with the mutation genotype of *APE1* Asp148Glu (Asp/Glu + Glu/Glu) showed thicker corneas, higher IOP and shorter axial lengths than those with the Asp/Asp wild-type genotype. Furthermore, there were thicker corneas, higher IOP and shorter axial lengths in carriers with the mutation genotype of *XRCC1* Arg399Gln (Arg/Gln + Glu/Glu) than those with the Arg/Arg wild-type genotype (all *P*<0.05) (Table 5).

**Table 5:** Correlation of gene polymorphisms of *hOGG1, APE1* and *XRCC1* with clinicopathological features of PACG patients

Clinicopathological features	*hOGG1* Ser326Cys		*APE1* Asp148Glu		*XRCC1* Arg399Gln	
	Ser/Ser	Ser/Cys + Cys/Cys	Asp/Asp	Asp/Glu + Glu/Glu	Arg/Arg	Arg/Gln + Glu/Glu
Gender						
Male	34	107	26	115	57	84
Female	24	93	18	99	46	71
Age (years)						
≤60	28	113	25	116	58	83
>60	30	87	19	98	45	72
Diseased eye						
Both eyes	26	90	16	100	46	70
One eye	32	110	28	114	57	85
Eyesight						
≤0.5	33	139	29	143	63	109
>0.5	25	61	15	71	40	46
Blood pressure						
Systole (mmHg)	141.9 ± 9.4	140.2 ± 8.2	143.0 ± 10.1	140.1 ± 8.2	141.5 ± 9.3	140.0 ± 8.2
Diastole (mmHg)	86.6 ± 6.7	86.0 ± 6.5	88.2 ± 8.0	85.9 ± 6.4	86.6 ± 7.0	85.8 ± 6.2
Eye condition						
Axial length (mm)	25.4 ± 8.2	21.9 ± 7.5*	26.1 ± 9.4	22.0 ± 7.5^†^	24.06 ± 7.4	21.3 ± 7.5^‡^
Corneal thickness (μm)	527.9 ± 7.3	547.6 ± 10.2*	523.3 ± 5.6	546.8 ± 10.1^†^	532.1 ± 6.8	550.7 ± 9.4^‡^
IOP (mmHg)	20.2 ± 5.3	25.4 ± 7.1*	20.1 ± 6.0	25.0 ± 7.0^†^	20.7 ± 5.2	26.6 ± 7.1^‡^

*, *P*<0.05 in comparison with Ser/Ser wild-type genotype; ^†^, *P*<0.05 in comparison with Asp/Asp wild-type genotype; ^‡^, *P*<0.05 in comparison with Arg/Arg wild-type genotype.

### Logistic regression analysis on the risk factors of PACG

A binary logistic regression analysis was conducted using PACG as the dependent variable and the Ser/Ser genotype of the Ser326Cys site, the Asp/Asp genotype of the Asp148Glu site, the Arg/Arg genotype of the Arg399Gln site, cornea thickness, IOP and axial length as the independent variables. As shown in Table 6, Asp148Glu and Arg399Gln polymorphisms could increase PACG risk (both *P*<0.05). It was also shown that Ser326Cys polymorphisms and cornea thickness had little influence on the occurrence of PACG, whereas a high IOP and short axial length are major risk factors of PACG (all *P*<0.05).

**Table 6:** Logistic regression analysis for the risk factors of PACG

Independent variable	B	S.E.M.	*P*	OR	95% CI
Ser326Cys	–0.383	0.275	0.164	0.682	0.397–1.169
Asp148Glu	–1.059	0.341	0.002	2.251	1.958–3.261
Arg399Gln	–0.859	0.295	0.004	1.635	1.226–3.183
Axial length	–0.844	0.093	0	1.782	1.563–2.377
Cornea thickness	0.019	0.016	0.231	1.019	0.988–1.051
IOP	1.138	0.225	<0.001	3.121	2.007–4.854

## Discussion

PACG is a major type of glaucoma in many Southeast Asian countries [19] and many PACG patients have similar anatomic features such as a shallow anterior chamber, increased lens thickness, anterior position of the lens, narrow anterior chamber angles and a short axial length [20]. Genetic factors have been documented to be associated with the development of PACG [21]. Genes involved in PACG susceptibility have been widely explored and the association between individual gene polymorphisms and PACG susceptibility has been noticed [20,22,23]. However, there are no reports on the association of *hOGG1, APE1* and *XRCC1* gene polymorphisms with PACG susceptibility and characteristic features, therefore, the current study was conducted.

The DNA repair enzyme system is important in maintaining the stability of a cell group and protects the cell genome from carcinogenesis by repairing damaged DNA. XRCC, XP and hOGG1 are common repair enzymes [24]. It has been found that the genetic diversity of repair enzymes affects both disease susceptibility and a tumour’s biological behaviour [25]. *hOGGl* is an important enzyme which removes 8-OH-G in DNA and has been found to possess SNP characteristics. Its gene mutation affects the enzymatic activity of hOGG1 and may lead to defects in DNA repair [26]. *hOGG1* Ser326Cys polymorphism reduces the DNA repair ability of hOGG1 proteins [27]. This may explain the association between *hOGG1* polymorphism and the elevated risk of PACG. Evidence shows that the *hOGG1* gene is especially important for *in vitro* DNA single-strand break repair and that the *in vitro* DNA-repair ability of the Cys/Cys homozygous genotype and Ser/Cys hybrid is significantly lower than that of the Ser/Ser wild-type genotype [28,29]. It has also been found that the cells with hOGG1-Ser326 protein expression are more effective in inhibiting mutations induced by 8-OH-G than hOGG1-Cys326. This indicates a relatively low repair ability of hOGG1-Cys326 in human cells [30]. Therefore, *hOGG1* Ser326Cys polymorphism lowers the DNA repair ability of the hOGG1 protein and increases the risk of PACG.

Base excision repair (BER) is the main DNA repair pathway that repairs damaged DNA bases caused by oxidative and alkylating reagents and plays an important role in the maintenance of DNA integrity [31,32]. APE1 is the key rate-limiting enzyme in the BER process and as a redox factor, can regulate the DNA-binding activity of transcription factors [33,34]. This is one mechanism that relates *APE1* Asp148Glu polymorphism to PACG susceptibility. *APE1* Asp148Glu is also a common *APE1* polymorphism site. Mutation of the site nucleotide Glutamic acid into Aspartic acid leads to increased chromosomal damage, reduces DNA repair ability and increases PACG susceptibility.

XRCC1 plays a critical role in BER [35]. Its polymorphic site (*XRCC1* Arg399Gln) is located in the binding domain of PARP (BRCT-1) and has a great affect on protein function. The mutation of Glutamine on the Arg399Gln site into Arginine leads to the mutation of amino acid Arginine in the 399th codon encoding into Glutamine. This reduces the DNA repair ability of *XRCC1* [36] and increases the risk of PACG. Previous studies have found that *XRCC1* gene diversity is related with the prevalence of nasopharyngeal carcinoma, laryngeal cancer and liver cancer [37–39]. It has also been demonstrated that *XRCC1* Arg399Gln is correlated with the incidence of the above-mentioned tumours and that allele Gln increases the risk of these tumours. Similarly, the present study also found that *XRCC1* Arg399Gln polymorphism is associated with the risk of PACG.

In summary, *hOGG1, APE1* and *XRCC1* gene polymorphisms are associated with the risk and characteristic features of PACG and therefore, can be used as biological indicators for PACG. However, there are limitations to our study. Glaucoma is a disease that involves many factors and multiple genes. The effect of various factors can easily be offset by another and lead to misleading results. Moreover, there are distribution differences among *hOGG1, APE1* and *XRCC1* gene polymorphisms in different regions. As the sample size is limited, it is necessary to carry out case-controlled researches in different ethnic groups, have larger sample sizes and use multi-factor analysis to further confirm our results.

## Abbreviations

APE1: apurinic endonuclease 1
BER: base excision repair
CI: confidence interval
dbSNP: Database of Single Nucleotide Polymorphisms
D’ value: disequilibrium value
hOGG1: human 8-hydroxyguanineglycosylase
IOP: intraocular pressure
OR: odds ratio
PAC: primary angle closure
PACG: primary angle closure glaucoma
PCR-SSP: PCR with sequence-specific primer
POAG: primary open angle glaucoma
SNP: single nucleotide polymorphism
XRCC1: X-ray repair cross-complementing gene 1
